# Cellular senescence links mitochondria-ER contacts and aging

**DOI:** 10.1038/s42003-021-02840-5

**Published:** 2021-11-24

**Authors:** Dorian V. Ziegler, Nadine Martin, David Bernard

**Affiliations:** 1 Centre de Recherche en Cancérologie de Lyon, Inserm U1052, CNRS UMR 5286, Université de Lyon, Centre Léon Bérard, Lyon, France; 2 Center for Integrative Genomics, University of Lausanne, Lausanne, Switzerland

**Keywords:** Senescence, Pathogenesis

## Abstract

Membrane contact sites emerged in the last decade as key players in the integration, regulation and transmission of many signals within cells, with critical impact in multiple pathophysiological contexts. Numerous studies accordingly point to a role for mitochondria-endoplasmic reticulum contacts (MERCs) in modulating aging. Nonetheless, the driving cellular mechanisms behind this role remain unclear. Recent evidence unravelled that MERCs regulate cellular senescence, a state of permanent proliferation arrest associated with a pro-inflammatory secretome, which could mediate MERC impact on aging. Here we discuss this idea in light of recent advances supporting an interplay between MERCs, cellular senescence and aging.

## Abstract (web summary)

This perspective by Ziegler et al. explores the impact of mitochondria-endoplasmic reticulum contacts (MERCs) biology on cellular senescence. The authors also explore the potential impacts of MERCs perturbation and how this relates to the increase in cellular senescence observed in common age-related diseases.

## Introduction

From the second half of twentieth century, the growing use of subcellular imaging through electron microscopy has allowed the identification of local physical contacts between multiple organelles and/or plasma membrane (PM), called membrane contact sites (MCSs), to the point that the field of “contactology” has emerged in the last decade^1–4^. Indeed, the first structural observations were extended to point out the multiple functional roles of these MCSs in regulating cellular responses in various pathophysiological conditions^4^. Firstly, MCSs constitute physical bridges to exchange metabolites (e.g. ions or lipids) between organelles, thus regulating intracellular metabolic fluxes. Secondly and as dynamic molecular microdomains, MSCs create subcellular signalling platforms, allowing local protein modifications and interactions^4^.

Endoplasmic reticulum (ER) and mitochondria constitute two key organelles involved in macromolecules synthesis and bioenergetics, but also in stress sensing and integration of cell fate signalling. As they form two independent networks occupying up to 45% of cell volume in eukaryotic cells^5,6^, ER and mitochondria are involved in multiple MCSs, including contacts between ER and PM, Golgi or peroxysomes, and between mitochondria and PM, lysosomes or lipid droplets^4^. ER and mitochondria are also able to interact with each other through mitochondria–ER contacts (MERCs)^7^.

**Fig. 1:**
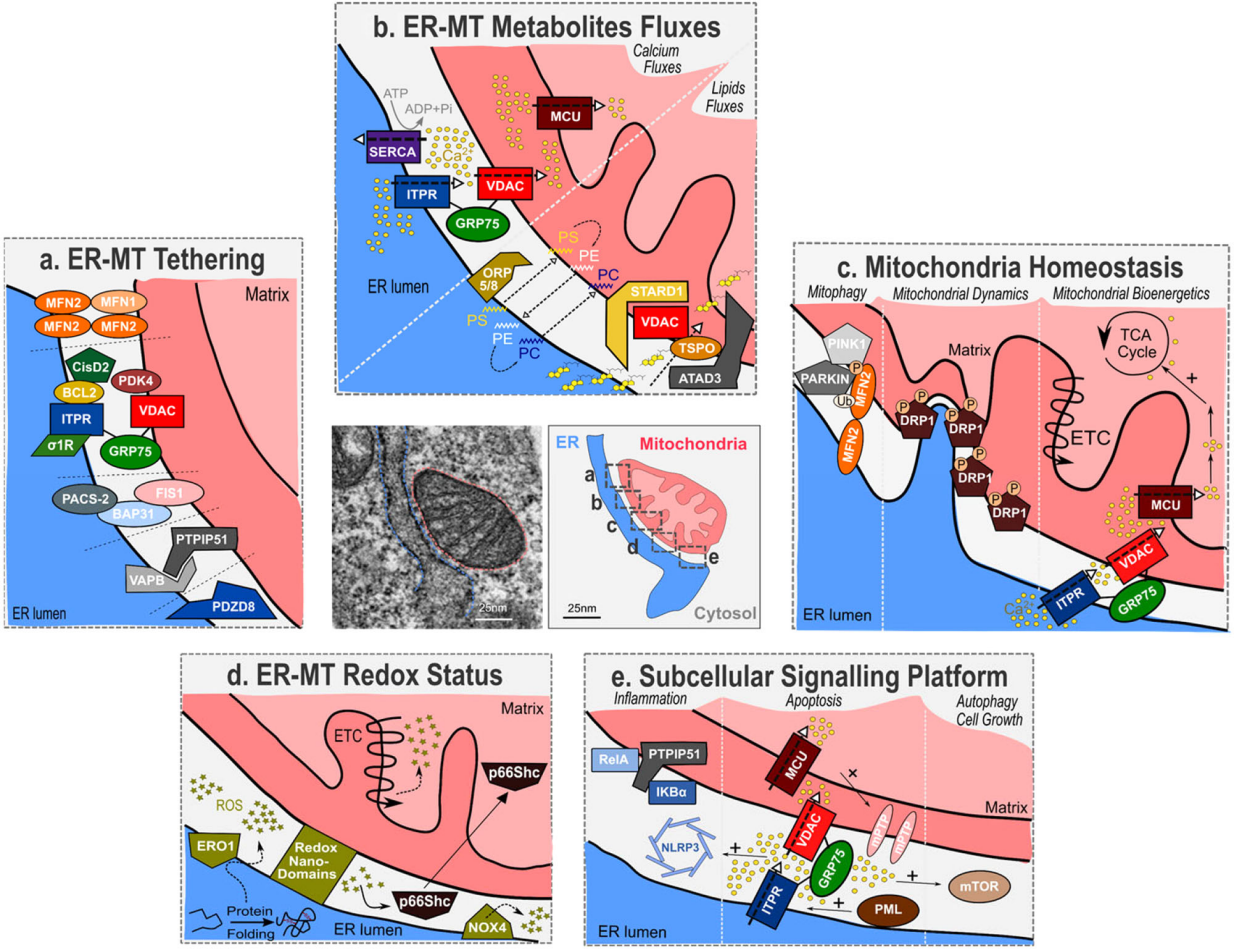
Key components and associated functions of mitochondria–ER contacts. Electron micrographs of MERCs, ER in blue and mitochondrion in red. **a** Multiple tethers allow the establishment of MERCs and include MFN1/MFN2 homodimers and heterodimers, ITPR-GRP75-VDAC complex, FIS1/BAP31, VAPB/PTPIP51 and PDZD8. Some MERC-associated proteins, including CisD2, PACS-2, PDK4 and SIGMAR1, interact with tethers to modulate MERCs. **b** MERCs are at the crossroad of calcium and lipids exchanges between ER and mitochondria. Dynamics of calcium fluxes within MERCs where ITPR, VDAC and MCU calcium channels insure transfer from ER to mitochondria and SERCA pump from cytosol to ER. Phospholipids (PS phosphatidylserine, PE phosphatidylethanolamine, PC phosphatidylcholine) are transferred trough MERCs, notably by ORP5/8 proteins, and cholesterol binds to STARD1/VDAC1/TSPO complex before being imported into the mitochondria. **c** MERCs regulate mitochondrial homeostasis notably through mitophagy, mitochondrial fission and mitochondrial bioenergetics. PINK1 phosphorylates MFN2, which recruits PARKIN at MERCs. PARKIN ubiquitinates MFN2 to initiate mitophagy. ER wraps mitochondria in initiation sites of fission and recruits DRP1. Calcium cation transfers from ER to mitochondria fuel some calcium-activated TCA cycle dehydrogenases and transporters to promote oxidative phosphorylation. **d** MERCs control redox status of ER and mitochondria. ERO1 is coupled to protein folding oxidation and generates ROS (H_2_O_2_). Redox nanodomains are formed within MERCs interface. NAPDH oxidase NOX4 produces ROS, while p66Shc senses ROS before relocalizing to mitochondria. **e** MERCs act as subcellular signalling platforms. Members of the NF-κB pathway, RelA and IKκBα, interact with PTPIP51. ITPR-mediated calcium release allows high local concentration of calcium and could participate in the recruitment and activation of mTOR and NLRP3. Promyelocytic Leukemia protein (PML) is found in MERCs fraction.

MERCs define tight contacts with a distance shorter than 50 nm specifically between ER and outer mitochondrial membranes^7^. MERC structures are dynamic structures known to be present in every eukaryotic cells, along approximately 10–15% of all mitochondrial membranes^2^. Highly heterogeneous among tissues and species, MERCs contain up to hundreds of proteins including tethering/structuring proteins, ion channels, transport binding proteins, enzymes and other signalling proteins^2^ (Fig. 1). Firstly described in the context of lipid biosynthesis/transfer, MERCs role has been largely extended to other metabolites fluxes, such as calcium^8–10^. MERCs are involved in lipid metabolism, calcium and redox signalling but also modulate mitochondrial dynamics^11^, autophagy^12^, inflammation^13^ and apoptosis^9^ (Fig. 1).

The use of genetic mouse models targeting MERC components has highlighted MERCs importance in controlling various pathophysiological situations, ranging from vascular remodelling to inflammation and metabolic disorders^13–17^. More recently, a role of MERCs in aging was suggested by evidences showing a modulation of their quantity and quality with specific age-related diseases or aging^18–20^. While autophagy and apoptosis are proposed to mediate some of MERCs age-associated effects^18,19^, whether other critical mechanisms are also involved remains unclear.

**Fig. 2:**
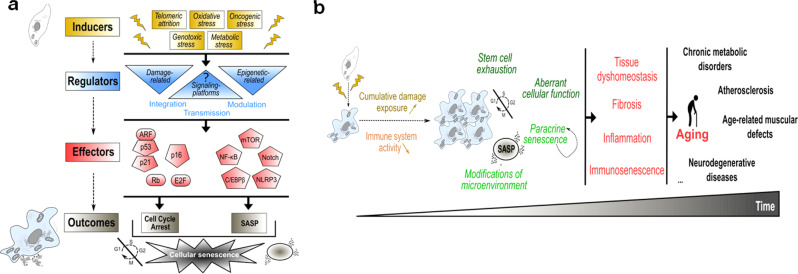
Cellular senescence: from regulation to involvement in aging. **a** Multiple stress signals arising from inducers (yellow) are sensed by regulators (blue) that include damage-related and epigenetic-related regulators. Signalling platforms may also act as main regulators. These interconnected regulators integrate, modulate and transmit senescent signals to the downstream effectors that include p53, p21, p16, Rb, E2F, NF-κB, C/EBPβ, mTOR, Notch and NLRP3 (red). These effectors ultimately trigger the main outcomes of cellular senescence (cell cycle arrest and SASP) (grey). SASP Senescence-associated secretory phenotype. **b** Over time, senescent cells accumulate in tissues due to increased cumulative damage exposure and reduced clearance (through decreased immune system activity). This accumulation leads to stem cell exhaustion, aberrant cellular responses, paracrine senescence (amplifying senescent cells accumulation) and modification of surrounding microenvironment. These cellular alterations lead to tissue dyshomeostasis, fibrosis, systemic inflammation and immunosenescence, finally contributing to aging and age-related pathologies.

Senescent cells accumulate during aging in various animal models^21,22^. Cellular senescence can be induced by a myriad of stresses and defines a state of permanent cell proliferation arrest and the concomitant acquisition of a senescence-associated secretory phenotype (SASP), including pro-inflammatory factors, pro-fibrotic factors and metalloproteases^23^ (Fig. 2a). Mechanistically, proliferation arrest is mediated by the activation of cyclin-dependent kinase inhibitors, mainly p21 and p16 (Fig. 2a). Besides, the SASP is mostly driven by NF-κB and C/EBPβ, and can be also positively regulated either by Notch, mTOR or NLRP3 pathways^24–27^ (Fig. 2a). Although these factors regulate cell cycle or SASP, the upstream molecular and subcellular mechanisms regulating them are largely unknown. Notably, signalling platforms mediating the link between stresses sensing and molecular activation of senescence effectors are largely unknown (Fig. 2a).

Cellular senescence has been early linked to aging, but the functional demonstration of its involvement in it was recent. The accumulation of senescent cells during aging is the concomitant result of both increased intracellular damage and declined senescence immune surveillance^28–30^ (Fig. 2b). While cell autonomous effects lead to stem cell exhaustion^31,32^ and aberrant cellular function^33^, non-cell autonomous effects through SASP can mediate paracrine senescence and modify the surrounding microenvironment. Subsequently, the chronic and systemic accumulation of senescent cells favours tissue dyshomeostasis, fibrosis, inflammation or immunosenescence, ultimately leading to aging and age-associated pathologies, including chronic metabolic disorders, atherosclerosis, age-related muscular defects and neurodegenerative diseases (Fig. 2b)^34–36^. Finding strategies to target and specifically eliminate senescent cells or attenuate their pro-inflammatory SASP, namely with senolytics or senomorphics, has thus become a major challenge in aging research field.

In this perspective, we will present and discuss recent advances suggesting a potential interplay of MERCs and cellular senescence in regulating aging. Based on the fact that MERCs and cellular senescence are both involved in aging and age-related pathologies, we compiled evidence that either modulation of MERC components or increased MERCs formation are pro-senescent signals. Lastly, we propose future directions to elucidate molecular mechanisms behind this emerging role of MERCs in regulating cellular senescence.

## MERCs and cellular senescence both control age-associated diseases and aging

While senescent cells accumulate during aging and participate in multiple age-related pathologies^21,37,38^, numerous clues indicate that MERCs could also play a role in age-associated diseases.

### Chronic metabolic disorders

Chronic metabolic disorders such as obesity and type 2 diabetes (T2D) account for two main contemporary diseases linked to overnutrition and aging^39^. High-fat diet (HFD) and obese pathological contexts promote senescence in several cell types and tissues including adipose tissue, liver, pancreas or brain promoting insulin-resistance and T2D^40–43^. Of note, a chronic increase of MERCs was observed in HFD-fed and *ob/ob* mice, two models exhibiting altered glucose homeostasis, insulin resistance and steatosis^44^. More strikingly, the use of an artificial MERCs linker^45^ is able to induce this insulin resistance^44^. Conversely, reduced MERCs in mice depleted for inositol-triphosphate receptor 2 (ITPR2) are able to improve glucose homeostasis, alleviating age-dependent steatosis and fibrosis^46^. Accordingly, decreased MERCs through *Pdk4* deletion ameliorates glucose homeostasis and insulin response^47^. Of note, decreased number of hepatic MERCs correlates with reduced mRNA and protein levels of p16 senescence marker^46^, known to be upregulated during aging^37,46^. Remarkably and as apparent contradictory result, decreased MERCs using genetic and pharmacological approaches targeting Cyclophilin D (CypD) also perturb insulin response in liver and muscle^48,49^. Taken together, these data demonstrate that an accurate balanced MERC number is necessary to maintain glucose homeostasis^15^.

Non-alcoholic fatty liver disease (NAFLD) is characterized by hepatic fat deposits, evolving from steatosis to fibrosis, cirrhosis and/or hepatocellular carcinoma^50^. As T2D, obesity and aging are three main factors of NAFLD, a detrimental role of cellular senescence has been proposed^51^. Nonetheless, recent functional studies highlighted the detrimental effects of some senolytics, namely Dasatinib and Quercetin, in the context of fully recapitulated NAFLD^52^, suggesting a complex role of cellular senescence in NAFLD. Furthermore, the elimination of p16^High^ senescent cells induces liver fibrosis if these senescent cells are not replaced^53^. In addition, senescence of hepatic stellar cells can inhibit their pro-fibrotic roles^28^, together showing different roles of senescent cells in liver diseases probably depending on the type of senescent cells and on their dynamic^54^. The importance of MERCs in NAFLD remains unclear^16^. For instance, *Mfn2* knockout was suggested to modulate MERCs quantity, though its precise role is still under debate^55^, and induces non-alcoholic steatohepatitis, one of the advanced stages of NAFLD, in mouse model. The importance of MERCs in the progression of NAFLD still needs to be further tested using specific MERC tools.

Altogether, all these data strongly indicate that MERCs disruption and modulation of cellular senescence are at the crossroads of age and obesity-related alterations of glucose metabolism and chronic liver dysfunction (Table 1).

**Table 1:** MERCs roles in senescence-associated age-related pathologies.

Roles		Cellular senescence		Mitochondria-ER contacts	
Methods		Functional studies		Functional and descriptive studies	
Age-related diseases		Beneficial	Detrimental	Beneficial	Detrimental
**Chronic metabolic disorders**		**Mice** → Functional (*p16* TG)^33^	**Mice** →Functional (SnCs clearance)^40–44^	**Mice** → Functional (*CypD* −/−)^48,49^ →Functional (*Mfn2* −/−)^16^	**Mice** →Functional (MERCs linker)^44^ →Functional (*Pdk4* −/−)^47^ →Functional (*Itpr2* −/−)^46^
**Atherosclerosis**		*—*	**Mice** →Functional (SnCs clearance)^38,58^	N/D	**In vitro** →Functional (*Pacs-2* silencing)^59,60^
**Age-related muscular defects**		*—*	**Mice** →Functional (SnCs clearance)^37^ →Functional (*p16* silencing)^63^	**Mice** →Functional (*Mfn2* −/−)^67,69^ →Functional (*CisD2* −/−)^68^	N/D
**Neuro-degenerative diseases**					
AD		*—*	**Mice** →Functional (SnCs clearance)^70,73^	**Rat** →Descriptive^80^ **Fly** →Functional (MERCs linker)^81^	**In vitro** → Descriptive^74^ **In vitro/Mice** →Descriptive^75^ **Fly** →Functional (*Pdzd8* silencing)^77^
PD			**Mice** →Functional (SnCs clearance)^71^	**In vitro** →Descriptive^82^ **Fly** →Functional (MERCs linker)^83^	**In vitro** →Descriptive^79^ **Fly** →Descriptive and functional (*Itprs* silencing/inhibition)^78^
FA			**In vitro**→Functional (*Frataxin* silencing)^84^	**In vitro** →Functional (*FxnFrataxin* silencing)^85^	N/D

		Beneficial	Detrimental	Beneficial	Detrimental
**Lifespan**		*—*	**Mice** →Functional (SnCs clearance)^37,180,181^	**Fly** →Functional (MERCs linker)^82^ **Worm** →Functional (*Grp75* knock-in)^97^	**Fly** →Functional (MERCs linker)^77^ **Mice** →Functional (*CypD* +*/−*)^94^ **Mice** →Functional (*Itpr2* −/−)^46^

Summary of the studies reporting a (beneficial or detrimental) role for MERCs and cellular senescence in age-related diseases and lifespan. For each study, the model is indicated in bold and experimental approaches, either descriptive (displaying correlations) or functional (modifying MERCs structure), are indicated underneath. *N/D* not determined, *AD* Alzheimer disease, *PD* Parkinson disease, *FA* Friedreich’s ataxia, *SnCs* senescent cells, *TG* transgenic.

### Atherosclerosis

Atherosclerosis is also promoted by overnutrition and aging, constituting the major cause of complications of cardiovascular diseases, including stroke and ischaemic heart failure^56^. Chronic atheromatous plaques are formed by endothelium dysfunction, the accumulation of oxidized low-density lipoprotein (oxLDL) in blood vessels and the subsequent aggregation of macrophages foamy cells and vascular smooth muscle cells (VSMCs). Endothelial cells, foamy cells and VSMCs in atheromatous plaques display senescence features^38,57^. The secretome of these senescent cells inhibits promigratory phenotype switching of medial VSMCs and their lesion entry for fibrous cap assembly^58^. Senolytics treatment rescues this promigratory phenotype-limiting atherosclerosis^38,58^. Interestingly, recent evidences pointed out that oxLDL treatment of VSMCs or endothelial cells impacts MERCs, notably thanks to PACS-2^59,60^, and mitochondrial calcium accumulation through increased MERCs is proposed to accentuate apoptosis of endothelial cells^60^. Nonetheless, sub-lethal elevation of mitochondrial calcium can also promote cellular senescence^61,62^. Taken together, and as correlations, these data suggest that early steps of atheromatous plaques formation involve MERCs modulation and cellular senescence (Table 1).

#### Age-related muscular defects

Muscular aging is characterized by reduced muscle fibre size and number, associated with loss of motoneurons, and terminally results in muscle dyshomeostasis. Muscular aging is driven at least partly by activation of senescence pathways in muscle stem cells through autophagy defects^63,64^ and mitochondrial dysfunction^65^. More importantly, the removal of senescent cells delays this age-associated muscular loss-of-function^37^. Noteworthy, aged muscle was reported to display a reduction of ER–mitochondria calcium fluxes coupling to higher mitochondrial oxidative stress^66^. Functionally, potential MERCs modulation through *Mfn2* knockout^55^ has detrimental effects in muscle and leads to age-related sarcopenia^67^. Furthermore, *CisD2* knockout mice also display premature muscle degeneration^68^. As age-related muscular defects may also come from neuromuscular synaptic loss, it is interesting to note that neuronal *Mfn2* is also reduced during aging and its specific deletion is sufficient to trigger skeletal muscle atrophy^69^. Overall, MERCs integrity or MERC components appear to participate in proper neuromuscular function, as MERCs uncoupling drives premature muscular aging (Table 1).

### Neurodegenerative diseases

Aging is considered as the main risk factor driving neurodegenerative diseases, which include among others Alzheimer disease (AD), Parkinson disease (PD), primary progressive multiple sclerosis (PPMS). While both neuronal and glial senescent cells accumulate during aging and lead to neural stem cells exhaustion, their elimination significantly ameliorates symptoms of AD, PD and PPMS^70–73^. MERCs were also studied in the context of neurodegenerative disorders. In AD patients, MERCs are enhanced and three enzymes involved in amyloid β generation (presenilin-1, presenilin-2 and γ-secretase) colocalize at MERCs^74,75^. Moreover, increased amyloid β induces expression of multiple MERC components including ITPR3 and voltage-dependent channel 1 (VDAC1)^75^, and presenilin-2 mutants display increased MERCs^76^. Importantly, *pdzd8* deletion decreases MERCs and rescues AD-associated locomotor decline in fly^77^. In PD, MERCs structure is altered^78^ and most of PD-associated proteins, such as α-synuclein, Parkin or PINK1, are found in MERCs fraction^79^. Furthermore, α-synuclein enhances MERCs and mitochondrial calcium uptake^80^. Taken together, these later studies suggest a detrimental role of MERCs in AD and PD. Noteworthy, multiple studies point out also decreased MERCs in PD- and AD-associated conditions, underlying also a beneficial role of these MERCs. For example, recent live FRET imaging of rat neurons displays decreased average length of tight MERCs in an AD model^81^. The use of an artificial linker in vivo also rescues locomotor defects in AD model in fly^82^. In a PD context, α-synuclein displays opposite role in MERCs formation as it can also decrease VAPB–PTPIP51 interaction^83^. Moreover, loss of parkin-induced ubiquitination of MFN2 is responsible for an inefficient coupling of MERCs and restoration of MERCs rescues motricity defects in a PD model in fly^84^. While no consensus, all these results support that MERCs dyshomeostasis could contribute to AD and PD. Finally, reduced MERCs are found in a model of Friedreich’s ataxia (FA), accompanied by decreased mitochondrial calcium and cellular senescence^85,86^, according to the importance of physiological basal mitochondrial calcium in sustaining mitochondrial bioenergetics^87^. Collectively, these data indicate that MERCs dysregulation and cellular senescence could be linked to neurodegenerative disorders. Nonetheless, while cellular senescence displays detrimental roles, MERCs roles are still under debate because of a low number of functional studies (Table 1).

### Systemic aging and lifespan

Senescent cells accumulate during aging in spite of multifactorial heterogeneity in the speed and level of their accumulation^88^. Removal of these senescent cells or reduction of their SASP result in a decrease of some age-related alterations as described above. Most importantly, this can also result in delayed aging and improve in both lifespan and healthspan^37,38,89–93^ (Table 1). Altogether, cellular senescence thus appears to be a key cellular phenotype driving tissue dysfunction and aging (Fig. 2b). Recent reviews have depicted how MERCs dyshomeostasis may be involved in some age-related pathologies and aging^18,19^. Among genetic mouse models studied in the context of MERCs dyshomeostasis, *CypD* knockout and *Mfn*2 knockout^67,94–96^ have been largely studied, while their specific role in MERCs is still under debate. Interestingly, lifespan of *CypD* haploinsufficient but not *CypD* knockout mice is increased compared to their control WT littermates^94^. Lifespan of *Mfn*2 knockout mice, although displaying premature muscular aging^67^, was not monitored. Noteworthy, *C. elegans* lifespan is extended by knock-in of *Hsp9a*, encoding the GRP75 (Glucose-Related Protein 75) scaffold protein binding to ER and mitochondria through ITPR and VDAC channels^97^. Though not clearly demonstrated, GRP75 may enhance MERCs in this model, and its muscle constitutive expression could counteract deleterious effects caused by MERCs disruption in muscle, as previously discussed^97^. More recently, the contribution of ER-mitochondrial calcium flux to aging in mice and worms was assessed. On one side, deletion of the murine ER-calcium release channel ITPR2 reduces MERCs and age-related alterations in males and females and it extends lifespan only in females^46^. On the other side, atf6 loss-of-function in worms results in sustained ITPR-mediated ER-mitochondria fluxes enhancing lifespan^98^. Altogether, these studies demonstrate that dyshomeostasis in both MERCs and ER-mitochondrial calcium fluxes may promote aging-associated phenotypes. Studies using genetic models targeting MERC components face major limits. For instance, whether *Mfn2* knockout reduces or increases MERCs number is still controversial^55,99–101^ even if it does not question the ability of MFN2 to contribute to MERCs. Furthermore, phenotypes driven by deletion of MERC components might be in some cases MERC-independent. As two major examples, MFN2 is associated to mitochondrial fusion^55^ and GRP75 (mtHsp70/Mortalin/GRP75) acts as a mitochondrial chaperone determinant for the quality control of intra-mitochondria folding of matrix-directed precursor proteins, these functions being independent of their roles in MERCs^102^. For these reasons, future works led on MERC components would need to clarify MERC-dependent and MERC-independent roles. In order to avoid MERC-independent roles, an alternative consists in using artificial linker/uncoupler of MERCs^2^. Whether forcing or uncoupling MERCs in vivo through artificial linkers/uncouplers may promote/delay aging and previously established age-related pathologies remains so far unclear. Two recent studies led on *Drosophila melanogaster* reported contradictory results on the same MERC-associated age-related pathology, namely AD. Using a synthetic linker, the two studies suggest either an extension^82^ or a reduction^77^ of lifespan. According to these results, while one reports that increasing MERCs ameliorates cognitive functions^82^, the other demonstrates that reducing MERCs has a similar effect, heightening mitophagy^77^. Clarifying the functional role of MERCs in physiological or pathological premature aging using specific linker/uncoupler in vivo is thus one avenue that would need to be explored in the future (Table 1).

Overall, these studies indicate that MERCs and cellular senescence can control similar age-related pathologies and establish multiple correlations between MERCs and cellular senescence in regulating aging (Table 1). That is the reason why the hypothesis of a role of MERCs in contributing to cellular senescence, which could at least partially mediate the impact of MERCs on aging, has recently emerged. Interestingly, numerous studies have reported that key MERC components are able to regulate features of cellular senescence.

## MERC components modulate key senescence features

MERCs are heterogeneous in terms of number and activity. MERCs composition is also highly variable according to recent proteomic data in different cell types^2,103–105^, challenging its specific study. Nonetheless, MERCs are regulated by abundance of scaffold proteins, defining MERCs quantity, and by other interface proteins, interacting with each other and including calcium channels, receptors and kinases, underlying MERCs activity and function. Noteworthy, quantity and activity/function of MERCs are interrelated as some enzymes and calcium channels, for example Pyruvate Dehydrogenase Kinase 4 (PDK4) and ITPRs, are able to regulate MERCs integrity^46,47,106^. Finally, numerous MERC components are regulated by protein interactions such as ITPR3-TOM70^107^, VDAC2-CDKAP4^108^, ITPR2-FUNDC1^109^, but also by post-translational modifications such as DRP1 SUMOylation by MAPL/MUL1^110^, which further adds a layer of complexity in the organisation and function of MERCs.

### Role of MERCs scaffold/tethering proteins in cellular senescence

MERCs establishment is regulated by multiple scaffold and tethering factors that include among others MFN2, GRP75-ITPRs-VDACs, FIS1-BAP31, VAPB-PTPIP51, PDZD8, CYPD and SIGMAR1^2,111^ (Fig. 1). MFN1 and MFN2 have been linked functionally to senescence^112^. MFN1 is a target of ubiquitin ligase MARCH5 and accumulates in MARCH5-deficient cells, which display hyperfused mitochondria and senescence features^113^. On the contrary, downregulation of *Mfn1* extends replicative lifespan^114^. Concerning *Mfn2*, its depletion boosts cellular proliferation of both B cell lymphoma cell line and mouse embryonic fibroblasts (MEFs)^115^. MFN2 mediates hyperfused mitochondria and promotes senescence of mesenchymal stem cells^116^. Concerning haematopoietic stem cells, opposite results highlight that *Mfn2* deletion induces defect in long-term lymphoid repopulation^117^, though no senescence markers were investigated. Overall, these data indicate that alterations of MFN1 and MFN2, key regulators of MERCs number, are able to regulate senescence-associated phenotypes. However, MFN1 and MFN2 are, independently of MERCs, also involved in mitochondrial fusion, and whether their effects on senescence depends on their function inside the MERCs is still unknown.

Concerning ITPR–GRP75–VDAC complex, GRP75 overexpression leads to increased replicative lifespan through downregulation of RAS and reduced phosphorylation of ERK2^118,119^ and ITPRs promote cellular senescence^46,61,62^, as it will be further discussed below.

BAP31 is an ER-resident chaperone transmembrane protein, found in MERCs fraction, forming a bridge with FIS1^120^ and known mainly to regulate apoptosis^121^. Although the functional implication of BAP31 has never been investigated in the context of cellular senescence, its deletion reduces cell proliferation of colon cancer cells^122^ and it is found to be upregulated in both replicative and X-ray induced senescence of fibroblasts^123^.

Overall, alterations of key MERC tethers are able to drive features of cellular senescence. Aside from these reports, further studies on additional tethers such as VAPB, PTPIP51 or PDZD8, or proteins participating in MERCs structure such as SIGMAR1 and CYPD, need to be conducted to determine whether they could also regulate cellular senescence.

### Role of MERC components involved in ER–mitochondria exchanges in cellular senescence

MERCs constitute a privileged site for exchanges of metabolites, including calcium and lipids, which have been recently involved in the regulation of cellular senescence.

Calcium exchanges at MERCs interface are regulated by multiple calcium channels, namely ITPRs (ITPR1, 2 and 3) in ER, VDACs (1, 2 and 3) at the OMM and MCU at the IMM for the mitochondrial influx and SERCA (1, 2) for ER influx (Fig. 1). At the ER interface, a few studies evaluated the impact of ITPRs in functionally regulating cellular senescence. ITPR2 knockdown results in the escape from oncogene-induced senescence (OiS) in human mammary epithelial cells (hMECs) but also delays replicative senescence in normal human fibroblasts^61^. *Itpr2* knockout MEFs display also a reduction of senescence markers associated with a reduced mitochondrial calcium accumulation over passages^46^. More strikingly, ITPR2 abrogation, which reduces some age-related alterations and increases mouse lifespan as described above, also lowers cellular senescence^46^. Concerning ITPR1 and ITPR3, their knockdown is also able to delay senescence^61^. Altogether these data point out a key role of ITPRs in the regulation of cellular senescence. Regarding VDAC1/2/3 or SERCA ATPases pumps, no studies reported their role in cellular senescence, in contrary to MCU, the IMM mitochondrial import calcium channel, which participates in OiS, probably through ITPR2-released calcium^61^. Importantly, decreased MERCs and deficient mitochondrial calcium uptake through depletion of frataxin lead to cellular senescence in neuroblastoma cells, highlighting also the importance of MERCs to ensure minimum ER–mitochondrial calcium fluxes^85,86^.

Besides calcium, MERCs constitute exchange sites for numerous other molecules such as phospholipid and cholesterol^2^ (Fig. 1). Oxysterol-Binding Protein-Related Protein 5 (ORP5) and 8 (ORP8) are ER-anchored proteins involved in phospholipids exchanges at MERCs. Strikingly, ORP5 administers phospholipid and calcium transfers, while ORP8 function is exclusively limited to phospholipid transfers. The constitutive expression of ORP5 boosts cell proliferation^124^, while its knockdown promotes senescence, through an increase of mitochondrial calcium uptake^125^. Of note, the sole downregulation of the exclusive phospholipid-exchanger ORP8 does not affect senescence^125^.

Taken together, the results suggest that MERCs calcium transfers could be predominant on phospholipid transfers in the regulation of senescence. Still, other functional studies on key MERCs proteins involved in the transfer of lipids between ER and mitochondria need to be performed in order to describe their potential contribution through MERCs in the regulation of cellular senescence.

Aside from tethers and transport proteins, multiple other MERCs proteins have been functionally involved in the regulation of cellular senescence, and especially of the SASP. Among SASP regulators, mTOR complex and inflammasome are respectively necessary for IL1-α translation and processing^25,26^. Interestingly, mTOR complex and NLRP3 are both found in MERCs^126,127^. Noteworthy, MERCs constitute hotspots for cytosolic calcium signalling and both mTOR and NLRP3 can be activated by calcium^128,129^. The hypothesis of a role for MERCs in calcium-mediated activation of mTOR and NLRP3, and subsequent SASP promotion, still needs to be tested. In addition to mTOR and NLRP3, two classical MERC resident proteins, namely PTIPT51 and PACS-2, are involved in the regulation of the NF-κB pathway which drives the pro-inflammatory SASP. While the MERCs tether PTPIP51 interacts with RelA and IKBα^130^, the induction of NF-κB programme upon irradiation in *Pacs-2* −/− thymocytes is abrogated, through blunted phosphorylation of IκBα, IκBβ and RelA^131^. Taken together, these data suggest that the mTOR, NLRP3 and NF-κB pathway could regulate SASP through MERC-dependent activation, even if more precise investigations should be conducted in the future to decipher this potential role.

p66Shc was largely studied in the context of senescence and also in relation with aging. Decreased p66Shc delays replicative senescence of human diploid fibroblasts^132^, and its increase promotes hepatocyte senescence and subsequent senescence-driven steatosis^133^. In the context of aging, p66Shc is phosphorylated at Serine 36 in an age-dependent manner in old animals, and strongly correlates with enhanced ROS production^134^. Though p66Shc intracellular localization is still under debate, frequently found in mitochondria, MERCs or PM-associated membranes, it has been suggested that p66Shc accumulates in MERCs with age before re-localizing to the mitochondria^134^. Exogenous oxidative stress is able to relocate p66Shc to the nucleus, where the oxidative stress response is integrated to establish a senescence response^135–137^. Collectively, these studies highlight the role of p66Shc as a sensor and regulator of cellular senescence and lifespan^135–137^.

Promyelocytic Leukemia protein (PML), an essential component of the PML nuclear bodies (PML-NBs), critically regulates cellular senescence^138^. Not only present in PML-NBs, PML is also located in cytoplasm, in ER and in MERCs^139^. PML limits phosphorylation of ITPR3, leading to dampened ER-mitochondria calcium flux and subsequent failed apoptosis^139^. A possible role of PML localization at MERCs in its pro-senescence function could be considered as well.

Two other MERCs proteins, CisD2 and PACS-2, were found to also regulate cell proliferation arrest, one of the key features of cellular senescence. CisD2, an ER protein located at the MERCs interface through Bcl2–IP3R interactions^140^, is necessary for proliferation of induced-pluripotent stem cells^141^. The knockdown of PACS-2, mentioned above for its ability to regulate NF-κB activation, also delays cell proliferation arrest induced by DNA damage–p53–p21 axis in thymocytes^142^. Additional cell types and complementary senescence markers should be monitored to better evaluate the contribution of CisD2 and PACS-2 in the modulation of the senescence fate.

Altogether these data indicate that several MERCs components, including tethers, calcium channels and others, regulate key features of cellular senescence (Table 2), although it still has to be proven that this action is MERC-dependent. Of note, it seems particularly interesting that some key SASP regulators are found associated and activated at MERCs interface. Overall, these data strongly suggest that MERCs regulate the senescence fate, which is an idea that has been recently evaluated.

**Table 2:** Regulation of senescence-associated features by MERC-associated proteins.

Function	Name	Effect of experiment on protein levels/activity (−/+)	Effect on MERCs number	Effects on ER and/or MT	Effects on senescence-associated phenotype	Ref.
ER-MT TETHERING	MFN1	+ (Stabilization)	N/D	Increased MT mass	Induction of senescence	^113^
	MFN2	− (KO)	N/D	N/D	Delayed RS	^115^
	GRP75	+ (OE)	N/D	N/D	Delayed RS	^118^
	FIS1	− (shRNA)	N/D	Hyperfused MT	Induction of senescence	^157^
		+ (OE)	N/D	Rescue hyperfused MT	Rescue of DFO-induced senescence	^156^
ER-MT METABOLITES TRANSFERS	ITPR1	− (shRNA)	N/D	N/D	Limited OiS	^61^
	ITPR2	− (shRNA)	N/D	Limited ER-MT calcium fluxes/ROS/MMD	Limited OiS and delayed RS	^61^
		− (siRNA)	N/D	Limited ER-MT calcium fluxes/MT ROS	Reduction of senescence	^62^
		− (KO)	Decreased	Limited ER-MT calcium fluxes/MT ROS/MMD	Reduction of senescence	^46^
	ITPR3	− (shRNA)	N/D	N/D	Reduction of OiS	^61^
	MCU	− (shRNA)	N/D	Limited ER-MT calcium fluxes	Reduction of OiS	^61^
	ORP5	− (siRNA)	N/D	Alteration of MT morphology and reduced OXPHOS	Induction of senescence	^125^
SIGNALLING PROTEINS	PACS-2	− (KO)	N/D	N/D	Resistance to p53-dependent CCA and NF-κB programme	^131,142^
	p66Shc	− (KO)	N/D	N/D	Delayed RS	^135^
	CISD2	− (KO)	N/D	Enhanced MMD	Reduction of cell proliferation	^141^
	RelA	− (shRNA)	N/D	N/D	Inhibition of SASP	^24^
	NLRP3	− (KO)	N/D	N/D	Reduction of age-dependent increase of p53 and p21	^27^
	mTOR	− (Rapa)	N/D	N/D	Inhibition of NF-κB-dependent SASP	^26^
	PML	− (KO)	N/D	N/D	Resistance to OiS	^138^
		− (KO)	N/D	Limited ITPR3 phosphorylation Reduced ER-MT calcium fluxes	N/D	^139^

Summary of the studies reporting a potential role for MERC-associated proteins involved in tethering, ER-MT metabolites transfers and signalling in the regulation of features of cellular senescence. For each study, the effect of MERCs protein dysregulation (upregulation + or downregulation−) on MERCs number and also ER and mitochondria are indicated. If investigated, the effects on features of cellular senescence are reported. *N/D* not determined, KO knockout, *OE* overexpression, *Rapa* Rapamycin, *ER* endoplasmic reticulum, *MT* mitochondria, *OXPHOS* oxidative phosphorylation, *MMD* mitochondrial membrane depolarization, *OiS* ocogene-induced senescence, *RS* replicative senescence, *CCA* cell cycle arrest.

## MERCs regulate cellular senescence

### A pro-senescent role for MERCs: potential mechanistic

Increased evidence of the role of MERCs proteins during aging and in cellular senescence raised the question of the importance of MERCs in regulating cellular senescence. Expression of an artificial linker tightening ER and mitochondrial membranes^45^ led to premature cellular senescence in normal human fibroblasts, with a NF-κB-dependent SASP^46^. This cellular senescence was accompanied by increased mitochondrial calcium and functionally involved ROS production, as antioxidant treatment rescued the onset of this premature senescence^46^. Downstream, p53 was necessary to induce senescence^46^. The importance of MERCs in established senescence models (replicative senescence, OiS, oxidative stress-induced senescence) should be critically tested in further investigations.

**Fig. 3:**
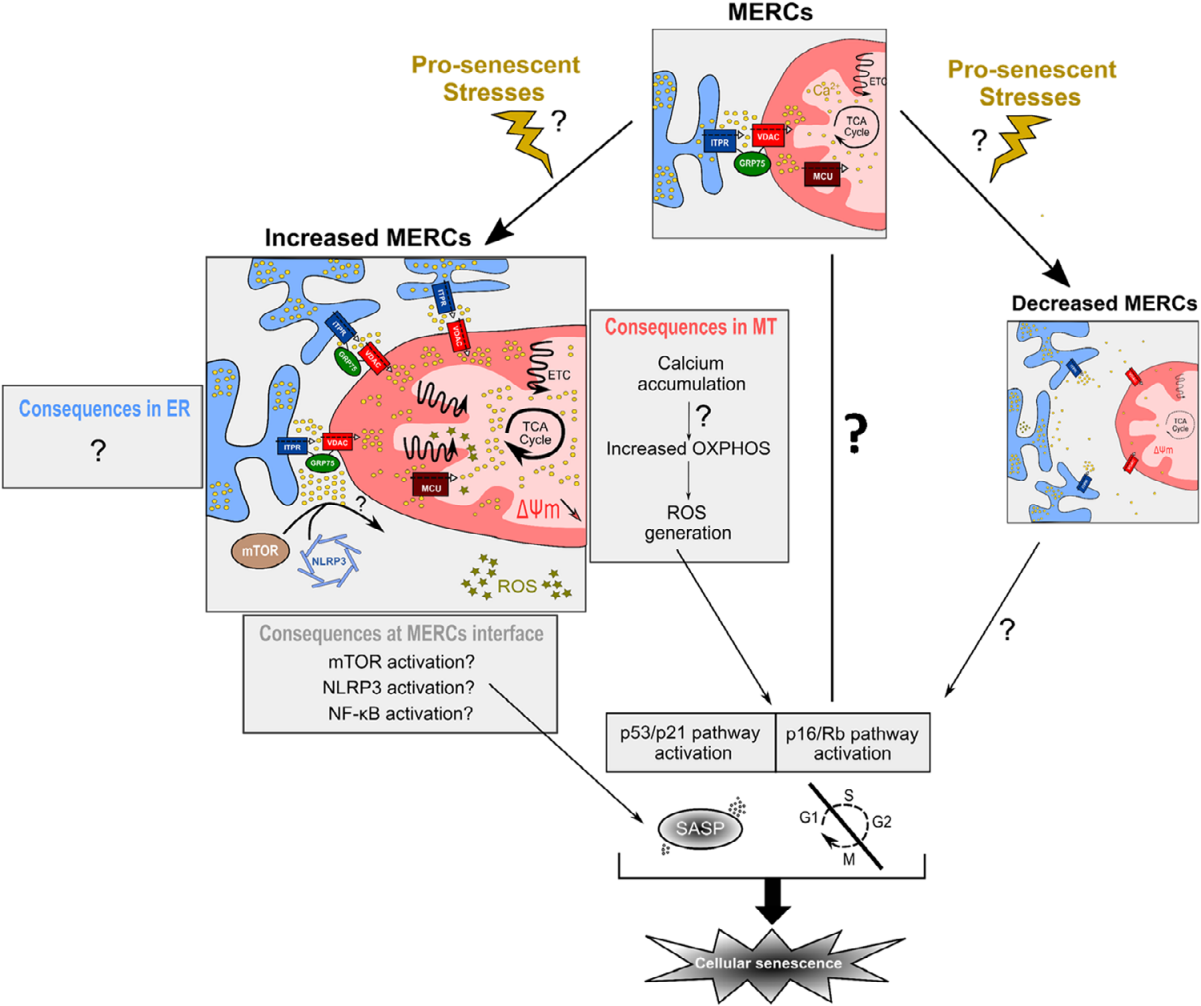
Mechanistic working model of MERC-induced senescence. Under physiological stimuli, MERCs allow proper calcium transfer from ER (in blue) to mitochondria (MT, in red). Upon pro-senescent stresses or other stimulations, MERCs number could be modified. Increased MERCs number (left panel) leads to mitochondrial calcium accumulation, a potential increased oxidative phosphorylation (OXPHOS) and an increased production of ROS activating p53/p21 and p16/Rb pathways to mediate cell cycle arrest and Senescence-Associated Secretory Phenotype (SASP), driven partly by NF-κB. In the meantime, whether increased MERCs would participate in ER-associated phenotypes, such as ER stress, is so far not known. In the cytosol-associated part, enhanced MERCs interface may activate three main SASP regulators, including mTOR, NLRP3 or NF-κB subunits. Finally, whether decreased MERCs number (right panel) may regulate cellular senescence and what could be the associated mechanisms remain to be critically addressed.

Noteworthy, deletion of MERC-associated proteins (such as MFN2, Frataxin or ORP5) and potential MERCs uncoupling were also proposed to mediate a senescence phenotype^85,86,113,125^, whether these effects are dependent of MERCs is unknown. To properly assess whether MERCs decrease may also impact senescence, it will be necessary to evaluate the impact of a specific MERCs spacer, such as FATE1^143^, in senescence models. While forcing MERCs induces a ROS- and p53-dependent senescence through an increased mitochondrial calcium uptake, an opposite role of MERCs uncoupling is nonetheless still unknown (Fig. 3).

How MERCs dyshomeostasis mechanistically drives cellular senescence remains an open question, but involves necessarily at least one of the three MERCs sub-compartments, i.e., mitochondrion, ER and apposed cytosol.

Mitochondrion side—An important number of studies report that senescent cells display mitochondrial abnormalities and various mitochondrial dysfunctions promote cellular senescence^112^. These mitochondrial dysfunctions are largely driven by non-exclusive mechanisms which include among others defective ETC, excessive ROS production, abnormal dynamics and altered mitophagy^112^. Nonetheless, the upstream mechanisms driving these mitochondrial dysfunctions during cellular senescence remain so far elusive.

In line with increased evidence of the role of calcium fluxes within the cell and specifically between ER and mitochondria^46,61,62,144,145^, calcium signalling appears to be an interesting candidate to mediate the senescence phenotype*.* In human endothelial cells and MEFs, replicative senescence is characterized by an increased MERCs number and a subsequent accumulation of mitochondrial calcium^46,146^. Accordingly, replicative senescent neurons display also an increased transfer of calcium from the ER to mitochondria accompanied by an upregulation of MCU expression^147^. Reduction of this mitochondrial calcium accumulation by knocking down ITPR2^61,62^ or MCU^61^ inhibits the establishment of cellular senescence in several cell models, including hMEC and fibroblasts^61,62^. When excessively accumulated, mitochondrial calcium leads to mitochondrial membrane depolarization, ROS generation and subsequent cellular senescence^46,61,62,144^ or apoptosis^148^. Of note, accumulation of mitochondrial calcium through the suppression of mitochondria calcium efflux by knocking down the mitochondrial Na^+^/Ca^2+^ exchanger triggers superoxide generation and neuronal apoptosis, driving AD-associated pathology^149^.

Mitochondrial calcium regulates mitochondrial bioenergetics mainly through its transfer through MERCs^87,150,151^. Indeed, mitochondrial dehydrogenases present EF-hand calcium binding necessary for enzymatic activity^152^. As mitochondrial calcium homeostasis is necessary in order to maintain mitochondrial bioenergetics^85,87,151,153^, massive MERCs uncoupling may also trigger mitochondrial dysfunction. For instance, reduced MERCs through *CypD* knockout^48,154^ leads to dysregulation of TCA cycle and fatty acid β-oxidation^95^, while no senescence markers were monitored in these studies. Interestingly, this dual role of the importance of a balanced ER-mitochondrial calcium influx has been recapitulated in the context of neural stem cell development in fly. Indeed, depletion of Miro, an OMM GTPase, reduces mitochondrial calcium and leads to mitochondrial metabolic impairment, whereas its constitutive expression triggers mitochondrial calcium overload and apoptosis^155^. Both conditions impaired neural stem cells lineage progression, though no senescence markers were investigated^155^.

To summarize the role of MERC-mediated mitochondrial calcium influx in cellular senescence, it appears that an altered calcium transfer from ER to mitochondria triggers mitochondrial dysfunction, eventually leading to cellular senescence. At this stage, most of the data support the hypothesis of an enhanced calcium influx into the mitochondria during cellular senescence, which could be mediated notably by increased MERCs, without excluding the hypothesis that reduced MERCs could also elicit pro-senescent signals (Fig. 3). Apart from calcium, whether lipid fluxes at MERCs interface are involved in mitochondrial alterations promoting cellular senescence remains an open question.

Beyond metabolites transfers, MERCs also promote early steps of mitochondrial fission through ER wrapping of mitochondria at future fission sites^11^. Reducing mitochondrial fission is able to induce senescence in normal cells^112,156,157^. Though no evidences were clearly established, MERC uncoupling could lead to accumulation of hyperfused damaged mitochondria by fission and subsequent mitophagy defects, eventually mediating a senescence phenotype. Nonetheless, whether the sole MERC uncoupling induces senescence features should be critically addressed (Fig. 3).

ER side—ER stress is a potent candidate to also mediate senescence phenotype^158^. This role is suggested by the fact that ER stress is observed in several models of senescence in vitro, such as OiS^159–162^ and UV- or X-ray-induced senescence^159,163^, but also in several senescence-associated pathological contexts, including therapy-induced senescence (TIS) in lymphomas^164^, diabetic nephropathy^165^, age-related sarcopenia^166^ and osteoarthritis^167^. ER stress may result from a persistent unfolded protein response (UPR) or from a luminal calcium depletion^168^ and includes three main sensors: PERK, ATF6 and IRE1^168^. Interestingly, MFN2 depletion in MEFs heightens the activity of the three ER stress branches^169^. Furthermore, PERK interacts with MFN2 and is found in MERCs fraction to transduce apoptosis triggered by ROS-mediated ER stress^170^. MFN2 is able to interact with PERK in normal conditions to restrain its activity. Remarkably, another ER-anchored MERCs tether, namely VAPB, interacts with ATF6, repressing transcription of its target genes^171^. Finally, IRE1 regulates lipid composition at MERCs and thus subsequent calcium fluxes^151^. Taken together, these data indicate cross-talks between MERC components and ER stress^169,170^. Concerning calcium fluxes, MERCs may decrease ER calcium content. ER calcium is determinant for chaperones involved in protein folding, such as Calreticulin or BiP/GRP78, and variations of ER calcium content subsequently lead to UPR and ER stress^172^. How ER stress is regulated upon modulation of MERCs and how it participates in cellular senescence should be addressed in the future.

Altogether, these data strongly suggest the importance of mitochondria in participating in the regulation of senescence phenotype, while ER contribution is still barely understood. Finally, some non-ER and non-mitochondria resident proteins modulating senescence features can be located in MERCs, as for example NLRP3, mTOR or PML. Therefore, MERCs could regulate cellular senescence by impacting signalling pathways in the cytosol through these proteins.

### MERCs: a dynamic platform for integrating pro-senescence signals?

MERCs are constantly modified and some pro-senescence signals were shown to affect MERCs protein levels. For example, persistent DNA damage response at telomeres during replicative senescence and X-ray-induced DNA damage upregulate the MERC tether BAP31 at mRNA levels^123^. Noteworthy, expression of ITPR2, the most efficient channel for ER-to-mitochondria calcium transfer^106^, is upregulated by other pro-senescence stresses, including oncogenic stress^61^, oxidative stress^173^ or high-fat diet^44^. Aside from transcriptional and translational regulations, some MERCs proteins were found relocated, as previously mentioned for p66Shc, or activated upon stresses inducing cellular senescence. Taken together, these data indicate that pro-senescence signals may modify MERCs composition and function. Proteomic analysis of MERCs during cellular senescence induced by different stresses will be needed to appreciate MERCs rearrangement in composition in response to pro-senescence signals.

MERCs quantity is also regulated upon senescence-inducing stresses. Increased MERCs were found during replicative senescence of endothelial cells and MEFs^46,146^. MERCs quantity may be regulated by redox stress, as, for example, antioxidant treatment rescues MERCs deficiency in FA model^85,86^. Importantly, DNA damage promotes the formation of MERCs to promote intrinsic cell death through enhanced mitochondrial calcium uptake^174^. At sublethal doses, oxidative stress and DNA damage also mediate cellular senescence^23^.

Altogether, it seems that MERCs are highly modulated in response to pro-senescence signals. Further studies need to be performed in order to evaluate how MERCs evolve during senescence in quantity, composition and function. Upon senescence-inducing stresses, MERCs could behave as platforms integrating these signals and modulating signalling to other subcellular compartments such as mitochondria, cytosol and nucleus where senescence features are regulated.

## Perspectives and conclusion

In conclusion, the observation that MERCs and cellular senescence play a crucial role in aging and age-associated diseases led to the hypothesis that MERCs may regulate aging at least partly through cellular senescence, and indeed, experimental data support that MERCs are key platforms controlling cellular senescence. This idea was first supported by studies showing a role for MERC components in cellular senescence and was strengthened by the observation that forcing MERCs with an artificial linker induces senescence in vitro. MERCs trigger this phenotype through ROS production and p53 activation. However how MERC-mediated mitochondrial calcium accumulation impacts mitochondrial functions and ROS generation and whether other calcium-independent processes are involved remains unknown.

Artificial linkers are powerful tools to investigate MERC functions but face some limitations. Some improvements have been obtained by the generation of inducible linker^175,176^ when compared to the constitutive artificial linker^45,46^. However, these linkers do not reflect many aspects of MERC complexity. Indeed, they do not allow to control the distance between ER and mitochondria (<50 nm but heterogeneous)^177^. This thickness is defined as the width of the cleft separating OMM from ER, and subdivides MERCs in tight (~10 nm) and loose (~25–40 nm) structures^178^. For instance, while loose MERCs were reported to promote ER–mitochondrial calcium transfers, tight MERCs were shown to limit them^177^. Beyond the distance between ER and mitochondria, the complexity of MERC composition and their plasticity critically orientate the effects of MERCs. There is then an urgent need to develop new tools to better manipulate MERCs to better understand the role of MERCs in controlling cellular senescence.

Whether MERC dysregulation is at the origin of cellular senescence which in turn results in pathological outcomes needs further investigations. For instance, the effects of linkers and spacers allowing to modulate MERCs need to be monitored in vivo, on cellular senescence and aging. Further studies will have to assess in these models and in in vivo models harbouring a knockout of MERC components (*Mfn2* −/−*, CisD2* −/−*, CypD* −/−*, Pdk4* −/−*, Pacs-2* −/−...*)* if the impact of these linkers, spacers and knockout on aging-associated alterations depends on cellular senescence. Crossing these models with models less prone to senescence or treating them with chemical compounds able to suppress features of senescent cells (senomorphics) or eliminate them (senolytics) would precise the importance of cellular senescence in the phenotypes induced by MERCs alterations.

We are then proposing that modulating MERCs could be a new avenue to modulate senescence, associated-pathological alterations and healthspan. Either the death of senescent cells could be promoted by increasing ER to mitochondria calcium flux or their accumulation could be reduced by lowering this flux. Chemical compounds targeting MERCs already exist^179^ and could be tested for these capacities.

In summary, we presented and discussed new insights into the potential role of MERCs in regulating cellular senescence which could participate in MERCs impact on aging. As it has been proposed for cellular senescence, we propose that the principle of antagonistic pleiotropy could be also applied to MERCs, which have a beneficial role for the cells when tightly regulated and which could be detrimental when dysregulated, leading to cellular senescence and associated consequences such as aging and age-related diseases. More broadly, this new field of research on the role of MERCs in cellular senescence and aging has highlighted the importance of communication between different compartments, sometimes mediated through contacts sites such as MERCs. This will pave the way to investigate the role of other MCSs, such as PM-ER, PM-MT or Lysosome-MT, in the context of cellular senescence and aging.
